# Plant Phenolics and Phenolic-Enriched Extracts as Antimicrobial Agents against Food-Contaminating Microorganisms

**DOI:** 10.3390/antiox9020165

**Published:** 2020-02-18

**Authors:** Miklós Takó, Erika Beáta Kerekes, Carolina Zambrano, Alexandra Kotogán, Tamás Papp, Judit Krisch, Csaba Vágvölgyi

**Affiliations:** 1 Department of Microbiology, Faculty of Science and Informatics, University of Szeged, Közép fasor 52, H-6726 Szeged, Hungary; kerekeserika88@gmail.com (E.B.K.); czambranocarrillo@gmail.com (C.Z.); primula15@gmail.com (A.K.); pappt@bio.u-szeged.hu (T.P.); csaba@bio.u-szeged.hu (C.V.); 2 MTA-SZTE “Lendület” Fungal Pathogenicity Mechanisms Research Group, University of Szeged, Közép fasor 52, H-6726 Szeged, Hungary; 3 Institute of Food Engineering, Faculty of Engineering, University of Szeged, Mars tér 7, H-6724 Szeged, Hungary; krisch@mk.u-szeged.hu

**Keywords:** phenolic antioxidants, antimicrobials, quorum sensing, biofilm, enterotoxin, foodborne pathogens, spoilage bacteria, carbohydrase aided extraction

## Abstract

Phenolic compounds and extracts with bioactive properties can be obtained from many kinds of plant materials. These natural substances have gained attention in the food research as possible growth inhibitors of foodborne pathogenic and spoilage bacteria. Many phenolic-enriched plant extracts and individual phenolics have promising anti-quorum sensing potential as well and can suppress the biofilm formation and toxin production of food-related pathogens. Various studies have shown that plant phenolics can substitute or support the activity of synthetic food preservatives and disinfectants, which, by the way, can provoke serious concerns in consumers. In this review, we will provide a brief insight into the bioactive properties, i.e., the antimicrobial, anti-quorum sensing, anti-biofilm and anti-enterotoxin activities, of plant phenolic extracts and compounds, with special attention to pathogen microorganisms that have food relation. Carbohydrase aided applications to improve the antimicrobial properties of phenolic extracts are also discussed.

## 1. Introduction

Phenolic compounds are secondary metabolites in plants and are considered as important natural molecules due to their bioactive properties. Phenolics are eliminators of free radicals and metal chelators. They can inhibit the lipid peroxidation and exhibit various physiological activities as antioxidants. In plants, these substances contribute to the defense mechanisms, and adaptation and pigmentation processes. Concerning human health, they have potential in the prevention and treatment of certain chronic diseases such as cardiovascular disease, diabetes and cancer [1]. In addition, plant phenolics and extracts rich in such substances can be excellent inhibitors of many foodborne pathogenic and spoilage bacteria [2,3]. Various food-related bacteria have biofilm-forming ability and can cause serious contaminations in the food industry. Quorum sensing, as a mechanism of bacterial cell-to-cell chemical communication, plays an important role in biofilm formation, antibiotic resistance, survival, proliferation and toxin production of the pathogens. Inhibition of this signaling process can contribute to the biological control of pathogenic organisms and bacterial toxins causing food deterioration and/or poisoning [4].

Byproducts of antioxidative plants generated through agro- and food industrial processes are excellent sources of bioactive phenolic materials with antimicrobial effects [5]. Physical and chemical extractions are common methods to obtain these active compounds from plants, but solid-state fermentation and enzyme assisted extraction procedures using carbohydrase active microorganisms or enzymes, can also be useful approaches [6,7,8].

Due to the growing knowledge on their bioactive values, use of plant phenolics as natural additives has recently gained interest in the food industry. Several studies demonstrated the potential of these antioxidant and antimicrobial compounds as food preservatives as well as functional food ingredients [9,10,11]. In this respect, phenolic acids, flavonoids and tannins have gained special attention over the last decades [2,12].

This review emphasizes the importance of using plant phenolics as natural alternatives of synthetic compounds to eliminate pathogens and spoilage bacteria from food environments. Also, this review deals on the inhibitory potential of phenolic antioxidants against the quorum sensing system, biofilm formation and enterotoxin production of food-related microorganisms. Some studies to improve the phenolic-related antimicrobial activity of plant extracts by using carbohydrases are also highlighted.

## 2. Foodborne Pathogens and Food Spoilage Organisms

Foodborne diseases constitute a global health problem. During the infection, pathogenic bacteria and/or microbial toxins produced enter to the human body through the contaminated food or water. Pathogenesis varies according to the host’s health conditions, the type of microorganisms and the amount of the agent to which the host is initially exposed. Common examples of food and waterborne outbreaks are the *Staphylococcus aureus* food poisoning and the *Salmonella* Typhimurium infection, which cause toxic symptoms and gastrointestinal infection. Table 1 presents some common foodborne pathogenic bacteria, their sources and produced toxins as well as the diseases caused.

Certain food pathogens can survive under adverse environmental factors such as cold, heat, acidic and high salt conditions and have the capacity to form biofilms on biotic or abiotic surfaces. These properties can facilitate their growth and spread on food contact surfaces as well.

On the other hand, the consumption of raw products, such as fruits and vegetables, packaged salads and ready-to-eat products has increased. This can cause diseases by exposing consumers to a greater variety of products potentially contaminated with food pathogens [17]. Moreover, the misuse and overuse of anti-infective drugs against pathogenic microorganisms has generated greater resistance to clinical antibiotic therapy, acquiring the ability to survive at high drug concentration that cause serious diseases and/or chronic infections [18,19].

Microbiological deterioration of foods adversely affects their physico-chemical properties and thereby the sensory characteristics. Texture softening, slime production, off-odors, off-flavors and colorization are the main signs of food spoilage. The main spoiling microbes in animal-derived products (e.g., milk, dairy, meat and poultry) are Enterobacteriaceae, lactobacilli, *Pseudomonas*, *Proteus* and *Brochotrix* species [20]. Yeasts and molds, i.e., *Saccharomyces*, *Candida*, *Pichia*, *Aspergillus*, *Penicillium*, *Botrytis* and *Fusarium* species, and bacteria such as pseudomonads, clostridia, bacilli and *Erwinia* are common spoilers in plant-derived products [20,21,22].

Prevention and control of foodborne pathogens and spoilers require their detection in the food. Conventional methods rely on culturing of microorganisms on special media allowing their identification and enumeration. These methods are precise but time- and labor-consuming. Some rapid detection method based on nucleic acids sequencing, metabolomics and proteomics have been developed in the recent decades [23,24]. Matrix-assisted laser desorption/ionization time-of-flight mass spectrometry (MALDI-TOF MS) can be used for the identification of pathogens through analysis of the whole cell proteome. Metabolites produced by pathogens or spoilers are detected by means of gas chromatography-mass spectrometry (GC-MS) or electronic nose [25]. Immunological methods, such as the enzyme-linked immunosorbent assay (ELISA), are also used for rapid detection of certain pathogens (e.g., *Salmonella*) [23]. For prevention and control of foodborne microorganisms, physical and chemical methods, like sterilization, pasteurization, irradiation, high hydrostatic pressure or preservatives can be used.

Some of these foodborne pathogenic and spoilage microorganisms became tolerant against the conventional food preservation and conservation methods [2]. There is a significant industrial demand for novel preservation techniques because of the common food losses due to microbial deterioration. Moreover, the consumers’ concern against the chemical preservatives in foods is growing, which also encourages the researchers to find natural alternatives with high antimicrobial potential. These substances then can be used as preservative agents to improve the shelf life of food products. Plant phenolic substances can be promising candidates for these studies.

## 3. Antimicrobial Activity of Plant Phenolics

**Figure 1:**
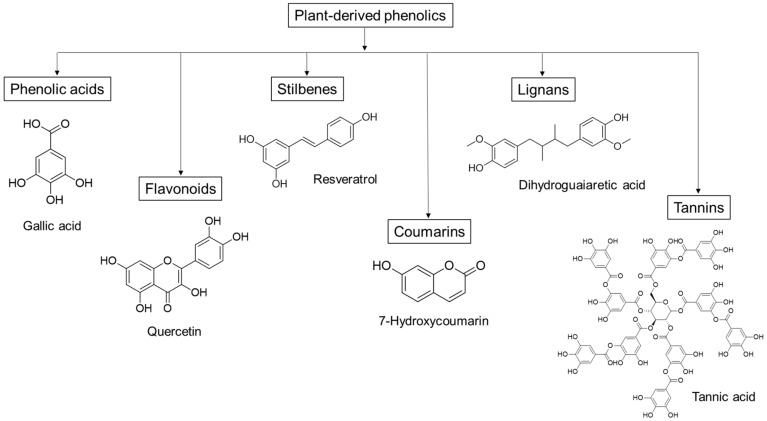
Groups of plant-derived phenolics, and some representative antimicrobial compounds with their chemical structure.

Plant-derived phenolics, such as phenolic acids, flavonoids, stilbenes and tannins, can inhibit the growth and activity of many microorganisms, including food-related pathogens as well as clinically important bacteria, fungi and protozoa [26,27,28]. Since the different molecules vary in their structure and chemical composition (Figure 1), they can display various antimicrobial effects, such as permeabilization and destabilization of the plasma membrane or inhibition of extracellular enzymes [29]. Moreover, these mechanisms of action differ from those of the traditional antibiotics, which could make plant phenolics effective against drug-resistant pathogens [29].

Extracts of grape pomace, grape seed, apple, and various exotic fruit and medicinal plant samples are frequently examined for their potential antimicrobial activity. In a pilot research, for instance, the influence of grape pomace extract on the growth of 14 pathogenic and spoilage bacteria was investigated [30]. In agar well diffusion tests, the order of the effective extract concentrations were found to be 20 > 10 > 5 > 2.5 > 1% (*w/v*), while in serial dilution assays, the sample at 0.5% (*w/v*) concentration had a bacteriostatic activity against *Escherichia coli* O157:H7 and *S. aureus*. In another study, red grape pomace possessed a strong bactericidal effect against *E. coli* and *S. aureus* at 12 mg/mL concentration [31]. The growth inhibitory effect, tested at concentrations from 0.5 to 2 mg/mL, varied in a dose dependent manner, and the *S. aureus* was more susceptible to the grape pomace extract than the *E. coli* strain tested. Grape seed extracts were also proved to be effective growth suppressors of other food-related bacteria such as *S.* Typhimurium, *Listeria monocytogenes*, *Bacillus* spp., *Pseudomonas aeruginosa* and *Campylobacter* spp. [32,33,34]. Among exotic fruits, extracts and betacyanin fractions of red pitahaya exhibited a good antimicrobial spectrum against Gram-positive and Gram-negative bacteria, yeasts and molds at concentrations from 7.8 µg/mL to 50 mg/mL [35,36]. Table 2 summarizes some additional examples about the antimicrobial activity of plant phenolic extracts, indicating the type of extraction and major antimicrobial activity indices.

Studies were also done using plant phenolic extracts as natural preservatives in food systems. The work of Sagdic et al. [58], for instance, was oriented towards in situ studies where they tested the antimicrobial activity of grape pomace extracts in beef patties. Samples from five grape varieties were incorporated to beef patties and the growth of Enterobacteriaceae, coliforms, *Salmonella*, *S. aureus*, total aerobic mesophilic count, yeast and molds, lactobacilli and micrococci pathogens was studied in different storage periods. Each pomace extract inhibited the microorganisms in a concertation dependent manner. Pathogenic bacteria, yeasts and molds were completely inhibited by 5 and 10% of the extracts. In another experiment performed in vegetable soup environment, the grape pomace extract showed antibacterial activity in a dose dependent manner against *S. aureus* and *E. coli*, due to its high proanthocyanidin content [59]. In meat paté, experiments of Hayrapetyan et al. [60] showed that a flavonoid-rich pomegranate (*Punica granatum*) peel extract could inhibit the growth of *L. monocytogenes* by 4.1 log at 4 °C during 46 days, but the inhibitory effect was less effective at higher temperatures (i.e., at 7 and 12 °C), demonstrating the influence of temperature on the inhibitory effect. Ahn et al. [61] added grape seed and pine bark extracts and oleoresin rosemary to ground beef and, after cooking, samples were inoculated with strains of foodborne pathogens (*E. coli* O157:H7, *L. monocytogenes*, *S.* Typhimurium and *Aeromonas hydrophila*). Pine bark extract at 1% concentration was the most effective against the growth of pathogens after 9 days of storage. Apart from the above studies, there were several experiments on the application of plant phenolic extracts as antimicrobials in various foods (these studies have recently been summarized by Bouarab Chibane et al. [62]).

Individual phenolic compounds of plant extracts have been shown to affect the growth of food-related microorganisms. In the study of García-Ruiz et al. [63], the antimicrobial activity of 18 phenolic compounds, i.e., hydroxycinnamic and hydroxybenzoic acids, stilbenes, flavan-3-ols, flavonols and phenolic alcohols, was evaluated against lactic acid bacteria wine isolates of *Oenococcus oeni*, *Lactobacillus hilgardii* and *Pediococcus pentosaceus*. Among the tested phenolics, the flavonols and stilbenes exhibited the strongest inhibitory effect on bacterial growth. In another experiment, Pastorkova et al. [64] investigated the antimicrobial potential of 15 phenolic compounds (i.e., phenolic acids, stilbenes and flavonoids) naturally occurring in grapes against wine spoilage yeasts and acetic acid bacteria. Pterostilbene, resveratrol and luteolin presented the major inhibitory effects on all tested microorganisms. Phenolic acids, i.e., myricetin, *p*-coumaric and ferulic acids, showed selective antimicrobial activity depending on the yeast and bacteria species tested. Activity of lignans and flavonoids were tested against *E. coli*, *L. monocytogenes*, *P. aeruginosa*, *Klebsiella pneumoniae*, *Enterobacter cloacae*, *S. aureus* and *Enterococcus faecalis* in the study of Favela-Hernández et al. [65]. Dihydroguaiaretic acid, 4-*epi*-larreatricin, 3′-Demethoxy-6-*O*-demethylisoguaiacin and 5,4′-dihydroxy-7-methoxyflavone compounds showed high growth inhibitory potential towards some of the tested strains, with a MIC range from 12.5 to 50 µg/mL. MIC values of 500–1000 μg/mL were reported for the 7-hydroxycoumarin (umbelliferone) against *S. aureus*, methicillin-resistant *S. aureus* (MRSA), *E. coli* and *P. aeruginosa* [66]. Table 3 shows other experiments from the last decade testing antimicrobial activity of individual phenolic compounds.

The phenolic compounds can express their microbicide effect through different mode of action. These molecules can suppress several microbial virulence factors (e.g., by inhibition of biofilm formation, reduction of host ligand adhesion and neutralization of bacterial toxins), reduce the fluidity of membrane, inhibit the synthesis of nucleic acids and the cell wall or energy metabolism [29,71]. In addition, many phenolics could show synergy with antibiotics enhancing their effectiveness and reducing the dose of use [72,73,74]. The presence and number of hydroxyl groups in phenolic compounds is responsible for their antioxidant properties. In addition, changes in the position of the hydroxyl group could play an important role in the antimicrobial activity [75] and the interactions with cell membrane structures [76]. In case of carvacrol, for instance, presence of the hydroxyl group and the delocalized electron system are thought to be responsible for the cytoplasmic membrane destabilization and the collapse of the proton motive force that finally led to death of *Bacillus cereus* cells [77]. Phenolic hydroxyl groups can form hydrogen bonds with active site of enzymes inhibiting their catalytic activity [78].

Antimicrobial action of phenolics may vary from molecule to molecule. In the study of Engels et al. [79], gallotannins isolated from mango kernel inhibited the growth of *Bacillus subtilis* and other foodborne pathogens such as *S. aureus* and *E. coli*. The inhibitory effects of gallotannins may be attributed to their iron-complexing properties and ability to interact with proteins and inhibit enzyme activities [79]. At the same time, flavonoids have a series of antibacterial actions with different mechanisms of action, such as inhibition of nucleic acid synthesis [80], induction of cytoplasmic membrane damage [81,82] and inhibition of energy metabolism [83], biofilm formation [3] and bacterial toxin production [84]. The flavonoid catechin can penetrate the lipid bilayers of the membrane resulting in leakage of intramembranous materials and liposome aggregation [85,86]. Moreover, in synergy tests, the catechin-rich fraction of green tea (*Camellia sinensis*) extracts could reverse the resistance to methicillin in MRSA [87].

On the other hand, it is possible to increase the antimicrobial activity of plant extracts by certain processes. An ecofriendly way is the treatment with carbohydrase enzymes that can hydrolyze the phenolic glycosides improving the antimicrobial potential of the samples. For instance, Pectinase 62L (10 U polygalacturonase equivalent activity) treatment for two hours at pH 5.0 and 37 °C caused a decrease in the minimum inhibitory concentration (MIC) values of bergamot peel extracts against *Salmonella enterica*, *Pseudomonas putida*, *E. coli* and *B. subtilis*, compared to the enzyme-free control [88]. The antimicrobial effect against different types of bacteria may depend on the enzyme cocktail(s) used for the treatment as well. In the study of Puupponen-Pimiä et al. [89], different pectinase treatments affected differently the inhibition potential of bilberry extracts against *Salmonella* and *Staphylococcus* bacteria. Pectinex Smash, Pectinex BE 3-L and Biopectinase CCM treated samples exhibited the highest antimicrobial activity against the *Staphylococcus* strains, while Pectinex Ultra SP-L, Pectinex 3 XL and Pectinex BE XXL treatments were superior in case of the *Salmonella* isolates. For pumpkin and flaxseed extracts, treatment using a mixed cocktail of immobilized *Aspergillus oryzae* α-amylase, and *Aspergillus niger* β-glucosidase and β-glucanase enzymes (in a ratio of 1:1:1) resulted in elevated antimicrobial activity against pathogenic bacteria compared to the enzyme-free sample [90]. Red grape pomace subjected to cellulase-assisted extraction efficiently inhibited the growth of *E. coli* and *S. aureus* pathogens in the study of Kabir et al. [31].

## 4. Quorum Sensing Systems and Biofilm Formation in Food Related Bacteria

The quorum sensing system is responsible for the formation of many food deterioration phenotypes [91]. The mechanism regulates important cellular functions such as biofilm formation, sporulation, expression of virulence genes, conjugation, competition, bioluminescence and the production of toxins and pigments. Since the quorum sensing is a density dependent communication mechanism, appearance of the controlled pathological events is related to the density of bacterial cells. The bacterial cells produce and secrete signal molecules, known as autoinducers that accumulate until reaching a sufficient local concentration of bacteria (a quorum) and start a series of population responses, including biofilm formation [92]. The autoinducers secreted by the Gram-negative bacteria are mainly N-acylhomoserine lactone (AHL) molecules (autoinducer-1). The autoinducers in Gram-positive bacteria are peptide compounds (i.e., autoinducer peptides, AIP). Furthermore, both Gram-negative and Gram-positive bacteria could secrete autoinducer 2 (AI-2), which are furanosyl borate diester and similar molecules. Other signaling factors, such as *p*-coumaroyl-homoserine lactone [93], unsaturated fatty acids [94] and alkylquinolones are also known [95]. Effective quorum sensing inhibition approaches could be the blocking of the synthesis and the secretion of the autoinducers, the enzymatic degradation of the signal molecules, and the receptor antagonism, in which the antagonist prevents the binding of the signal molecules to response regulator proteins (e.g., to LuxR). However, according to recent investigations, resistance could be developed against certain anti-quorum sensing treatments [96]. Furanones are well-known natural antimicrobials that show destructive activity against the quorum sensing system of both Gram-negative and Gram-positive bacteria [97,98].

Biofilms are microbial communities attached to biotic and abiotic surfaces and embedded in a matrix of extracellular polysaccharides, lipids, proteins and nucleic acids, the so called extracellular polymeric substances (EPS) that are produced by the microbial community itself. Inside the biofilm, the cells display different metabolic activity and physiological, gene expression and morphological patterns compared to the planktonic cells. They become more resistant to environmental adverse factors, such as the lack of nutrients and oxygen and changes in the pH condition. Biofilm bacteria are less sensitive to the action of antimicrobial agents causing a potential risk in food industry environments [99]. In addition, the antimicrobial substances at subinhibitory concentration can act as environmental signals activating the formation of biofilms [100], thereby, leading to the failure of the drug treatment [101]. However, an effective quorum sensing inhibitor could inhibit the biofilm formation of foodborne pathogenic and spoilage bacteria as well [102,103,104]. Therefore, in agreement with today’s consumer demands, there are food preservative developments focusing on the screen and extensive analysis of natural inhibitory systems.

## 5. Anti-Quorum Sensing and Antibiofilm Effects of Plant Phenolics

Certain plant phenolic substances, including phenol-rich crude extracts, could exhibit anti-biofilm and/or anti-quorum sensing activities [105,106,107]. The phenolic compounds suppress the bacterial biofilm formation by the inhibition of different regulatory mechanisms without affecting growth: they can block the quorum sensing as mentioned above, reduce the bacterial motility altering their performance [108], decrease the superficial adhesion [109] and inhibit the expression of virulence factors associated with pathogenic behaviors [110,111].

In the study of Vattem et al. [112], the anti-quorum sensing effect of aqueous phenolic extracts from common dietary fruits, herbs and spices was investigated, using the purple pigment violacein producer *Chromobacterium violaceum* CVO26/CV 31532 bioassay system. The violacein synthesis is under quorum sensing regulation, mediated by AHL autoinducers. Among the fruit extracts tested, raspberry, blueberry and grape samples inhibited the AHL activity, and blueberry had the highest effect on the AHL synthesis. Moreover, blueberry extract was outstanding in the inhibition of quorum sensing related swarming motility in *P. aeruginosa* and *E. coli* O157:H7 pathogens. Berry phenolic extracts, namely those from raspberry and cloudberry, were the most effective *C. violaceum* AHL signaling inhibitors in the study of Priha et al. [113]. In addition, the cloudberry extract reduced the biofilm formation of the common brewery contaminant bacterium, *Obesumbacterium proteus* at the concentrations of 25 and 50 mg/L. A bioactive phenol-rich extract from apple peel was also tested for its anti-quorum sensing effect in the *C. violaceum* agar-diffusion test system by Fratianni et al. [38]. The whole extract exhibited quorum sensing inhibiting activity, which, however, was not detected for the single phenolic compounds of the apple peel. Here, the authors pointed out on possible synergistic or combinatory effects between the molecules in the extract, resulting in anti-quorum sensing activity for the crude sample. In a *C. violaceum* based liquid test, significant inhibition of violacein production was recorded for syringic acid, vanillic acid, (+)-catechin and resveratrol compounds (10 µg/mL), that can be found at different concentrations in black grape, apple and pitahaya extracts [3]. The yield of some of these phenolics, obtained via carbohydrase-assisted extraction, showed positive association with the anti-quorum sensing activity of the crude extracts tested. In the same research, all single phenolic compounds tested, i.e., 4-hydroxybenzoic, syringic, gallic, vanillic, cinnamic and *p*-coumaric acids, (+)-catechin, (−)-epicatechin, quercetin, polydatin and resveratrol, inhibited the biofilm formation of *L. monocytogenes*, *S. aureus*, MRSA, *E. coli*, *S. enterica*, *P. putida* and *P. aeruginosa* pathogens *in vitro*, at 100 µg/mL concentration [3].

In addition, many other studies have addressed the ability of phenolic compounds and different plant extracts to modulate the quorum sensing system and the biofilm formation in foodborne pathogenic and spoilage bacteria. For instance, catechin [114], naringenin [115] and quercetin [116] depicted strong anti-quorum sensing property against *P. aeruginosa*. Additionally, flavonoid fraction of guava (*Psidium guajava* L.) leaves extract inhibited the quorum sensing system of *C. violaceum*, and the biofilm formation, pyocyanin production, proteolytic and elastolytic activities and swarming motility in *P. aeruginosa* PAO1 [117]. It was shown that the quercetin and quercetin-3-*O*-arabinoside components of the flavonoid extract were responsible for the anti-quorum sensing activity.

There were several investigations concerning the inhibition of pathogenic *E. coli* biofilms by phenolics. In the study of Lee et al. [118], the flavonoid phloretin, a major compound in apple and strawberry extracts, has inhibited the formation of *E. coli* O157:H7 biofilms without affecting the growth of planktonic cells. Two furocoumarins isolated from grapefruit juice, bergamottin and dihydroxybergamottin, suppressed the biofilm formation of *E. coli* O157:H7 in a range of 71.9 and 58.3%, respectively [119]. Furthermore, naringenin, quercetin, sinensetin and apigenin were effective quorum sensing antagonists and biofilm suppressors in *E. coli* O157:H7 strain [120]. For non-O157 Shiga toxin producing *E. coli* strains, Sheng et al. [121] found that the grape seed extract inhibited well the quorum sensing system.

Many phenolic acids proved to be effective against *S. aureus* biofilms as well. With this context, the gallic [122], ginkgolic [123], ellagic [124] and rosmarinic acids [125] have been found to be promising inhibitors in the research of the past decade. The phenolic glycoside compound, 1,2,3,4,6-penta-*O*-galloyl-β-D-glucopyranose, purified from *Eustigma oblongifolium* extract, inhibited the formation of *S. aureus* biofilms by blocking the synthesis of cell-to-cell adhesion compounds, thereby, preventing the primary attachment to solid surfaces [126]. It was also shown that methanol extract from pomegranate, rich in ellagic acid, inhibited the biofilm formation of *S. aureus*, MRSA, *E. coli* and *C. albicans* [124]. Red wines, extensively recognized for their high flavonoid (e.g., quercetin, kaempferol, apigenin, chrysin, fisetin and luteolin) and stilbenoid (e.g., *trans*-resveratrol) content have proven to be potent inhibitor of *S. aureus* biofilms [127]. Among the compounds tested, the quercetin exhibited the highest biofilm inhibitory potential. In addition, phenolic extracts from muscadine grape were also able to inhibit and eradicate the *S. aureus* biofilm in the study of Xu et al. [128]. A summary of some recently published researches about antibiofilm activity of plant extracts against food-related microorganisms is presented in Table 4.

## 6. Anti-Enterotoxin Effect of Plant Phenolics

Many phenolic compounds and extracts even at concentrations below the MIC can inhibit the production and/or the activity of bacterial enterotoxins [139]. These anti-enterotoxin properties are being intensively tested for foodborne pathogens, especially in case of *S. aureus*. The staphylococcal enterotoxins and enterotoxin-like molecules are low-molecular weight proteins with a globular structure. They have superantigenic activity and are varied in their emetic potential [140]. Among them, the enterotoxin A is responsible for most staphylococcal food poisoning outbreaks [141]. Phenolic compounds can affect the enterotoxin production through several mode of action, including translation and/or transcription inhibition, disruption of secretory mechanisms, inhibition of quorum sensing regulatory systems, and toxin inactivation [142,143]. Various plant derived phenolic substances have been described as effective inhibitors of the staphylococcal enterotoxin production and activity (Table 5).

Activity of phenolics on AB-type protein toxins, e.g., cholera toxin, Shiga toxins, *E. coli* heat-labile toxin, *P. aeruginosa* exotoxin A, has also been extensively studied. These toxins consist of an A catalytic subunit and a B cell-binding subunit. Grape extracts inhibited the cholera intoxication in cultured cells and intestinal loops through various actions, including the elimination of the pre-bound toxin from the cell surfaces, and disruption of the unfolding, transport and catalytic activities of the dissociated A subunit [152]. In a later study, the function of 20 individual phenolic constituents of grape extracts in cholera toxin inhibition was assessed [153]. Among others, inhibitory functions affecting the toxin binding and the enzyme activity have been associated with the mode of action of individual phenolic compounds. For instance, resveratrol disrupted the toxin internalization and activity, epigallocatechin gallate and procyanidin blocked the toxin binding and occupied the binding sites, and kaempferol and quercitrin could directly inhibit the activity of the catalytic subunit. Grape seed and grape pomace extracts effectively disrupted the action of the Shiga toxin 1 and 2, and the heat-labile toxin as well [152,154].

## 7. Conclusions

Many phenolic compounds and phenol-rich plant extracts have promising activity to inhibit the growth of both the planktonic form and the biofilms of food related pathogens. Investigation of this property is particularly important as bacterial biofilm layers are commonly formed on foods and/or food contact surfaces, resulting in a microbial community more resistant to the traditional disinfectant agents. Moreover, their inhibitory properties against the production and activity of bacterial enterotoxins can make many plant phenolics effective in preventing food poisoning symptoms. Plant phenolics could have anti-quorum sensing activity as well. The quorum sensing mechanism regulates the biofilm formation and toxin production of pathogenic bacteria; therefore, discovery and analysis of substances suppressing this system has also occupied a prominent field in the current researches. In conclusion, the summarized studies emphasize not only the importance of plant phenolic extracts as sources of natural preservatives but provide alternatives for ecofriendly utilization of some agro- and food industrial byproducts and enzyme aided extraction processes as well.

## Figures and Tables

**Table 1:** Most common foodborne pathogenic bacteria, their produced toxins and diseases caused.

Foodborne Pathogen Bacteria	Toxin Production	Type of Disease	Main Food Sources of Infection	Reference
*Bacillus cereus*	Emetic toxin, diarrheal toxin	Emetic syndrome, diarrhea	Rice, pasta, noodles, pastry	[13]
*Campylobacter coli*, *Campylobacter jejuni*	Cytolethal distending toxin	Campylobacteriosis	Poultry products, unpasteurized milk	[14,15]
*Clostridium botulinum*	Botulinum toxin	Botulism	Improperly processed canned foods	[13]
*Escherichia coli* O157:H7	Shiga-toxin	Hemorrhagic colitis	Ground meats, raw or under-pasteurized milk, sprouts	[13,15]
*Listeria monocytogenes*	Listeriolysin O	Listeriosis	Soft cheeses from unpasteurized milk, ready-to-eat products	[15,16]
*Salmonella* Typhi, *Salmonella* Typhimurium, *Salmonella* Enteritidis	Enterotoxin	Typhoid fever, salmonellosis (gastroenteritis)	Any type of food: meat, poultry, fish, milk, eggs, vegetables, water	[13,15]
*Staphylococcus aureus*	Heat stable enterotoxins	Gastrointestinal symptoms	Meat, dairy products, salads	[13]
*Vibrio cholerae*, *Vibrio parahaemolyticus*, *Vibrio vulnificus*	Cholera toxin	Cholera, gastroenteritis	Raw/undercooked shellfish, meat, contaminated water	[13]

**Table 2:** Antimicrobial activity of plant phenolic extracts, some examples.

Plant Materials	Type of Extraction	Target Organism	Antimicrobial Activity	Reference
**Fruit samples**				
Red wine grape pomace	70% acetone/0.1% HCl/29.9% water (*v/v/v*)	*Escherichia coli*, *Listeria innocua*	Pinot Noir-pomace and skin MIC ^1^: *E. coli*, 3% and 6%; *L. innocua*, 2% and 7%	[37]
			Merlot-pomace and skin MIC: *E. coli*, 9%; *L. innocua*, 8%	
Apple peel	Ethanol	*Lactobacillus acidophilus*, *Lactobacillus bulgaricus*, *Lactobacillus plantarum*, *Lactobacillus rhamnosus*, *E. coli*, *Bacillus cereus*, *Staphylococcus aureus*	Extract concentration: 2-20 µg/disk	[38]
			*B. cereus* and *E. coli*: inhibition haloes of 6 to 14 mm	
			Probiotic lactobacilli: no inhibition	
			*S. aureus*: no inhibition	
Red and white grape pomaces	Acetone	*Listeria monocytogenes*, *S. aureus*, *E. coli* O157:H7, *Salmonella* Typhimurium	MIC (mg/mL): *L. monocytogenes*, 4.69–18.8; *S. aureus*, 40.6–250	[39]
			MBC ^2^ (mg/mL): *L. monocytogenes*, 9.38–37.5; *S. aureus*, > 250	
			*E. coli*: no inhibition	
			*S.* Typhimurium: no inhibition	
Cinnamon bark and Ajowan fruit	Acetone, ethanol	*Pseudomonas sp.*, *Bacillus subtilis*, *E. coli*, *S. aureus*	MIC values (μg/mL):	[40]
			ethanol extract of cinnamon, 32–64; ethanol extract of Ajowan, 32–64; acetone extracts of cinnamon, 16–64; Acetone extract of Ajowan, 64–128	
Blackberry and blueberry pomaces	10% methanol, 10% ethanol	*Campylobacter jejuni*	MIC (mg/mL GAE): blackberry, 0.6; blueberry, 0.4	[41]
			MBC (mg/mL GAE): blackberry, 0.8; blueberry, 0.5	
Blueberry puree	75% ethanol	*L. monocytogenes*, *Salmonella* Enteritidis	MIC (mg/mL): *L. monocytogenes*, 300–750; *S.* Enteritidis, 400–1200	[42]
Apple pomace	Ethyl acetate	*S. aureus*, *E. coli*	MIC (mg/mL): *S. aureus*, 1.25; *E. coli*, 2.50	[43]
Black grape pomace, apple and pitahaya residues	10% ethanol after enzyme-aided extraction	*B. subtilis*, *B. cereus*, *L. monocytogenes*, *S. aureus*, methicillin-resistant *S. aureus*, *E. coli*, *S.* Typhimurium, *Pseudomonas putida*, *P. aeruginosa*	MICs: from 12.5 to ≥ 100 mg/mL	[3]
Bayberry	Ethanol	*S. aureus*, *L. innocua*, β-hemolytic *Streptococcus*, *S.* Enteritidis, *Salmonella typhi*, *Shigella dysenteriae*	Diameter of inhibition (mm):	[44]
			*S. aureus*, 22.9; *L. innocua*, 21.5; β-hemolytic *Streptococcus*, 22.7; *S.* Enteritidis, 20.1; *S. typhi*, 13.3; *S. dysenteriae*, 19.3	
Grape pomace	50% methanol, 50% ethanol	*S. aureus*, *E. coli*, *P. aeruginosa*, *Candida albicans*	Extract concentration: 1 mg/disk	[45]
			Diameter of inhibition (mm):	
			*C. albicans*, 12–13	
			*S. aureus*, *E. coli*, *P. aeruginosa*: no inhibition	
Grape residues	Ultrasound-assisted extraction, methanol:acetone: water:acetic acid (30:42:27.5:0.5)	*Clostridium perfringens*, *B. cereus*, *L. monocytogenes*, *S. aureus*, *Sarcina lutea*, *Micrococcus flavus*, *E. coli*, *P. aeruginosa*, *S.* Enteritidis, *Shigella sonnei*, *Klebsiella pneumoniae*, *C. albicans*	Extract concentration: 30 µg/disk	[46]
			Diameter of inhibition (mm):	
			*C. perfringens*, 15.9–17.7; *B. cereus*, 15.2–17.1; *L. monocytogenes*, 16.4–18.5; *S. aureus*,16.5–18.5; *S. lutea*, 17.3–19.7; *M. flavus*, 14.8-16.9; *E. coli*, 12.1–15.7; *P. aeruginosa*, 13.5–15.9; *S.* Enteritidis, 13–15.4; *S. sonnei*, 15.6–17.7; *K. pneumoniae*, 15–16.6; *C. albicans*, 13.1–15.5	
Grape marc waste	Aqueous extraction and Amberlite FPX-66 purification	*E. coli*, *S. aureus*, *C. albicans*	MBC (%, *w/v*): *E. coli*, 2; *S. aureus*, 0.125; *C. albicans*, no effect	[47]
Apple phenolic fractions	Acetone:ethanol (1:3), solid phase extraction	*L. monocytogenes*, *S. aureus*, *E. coli*, *S.* Typhimurium	Extract concentration: 10–5000 µg/disk	[48]
			Diameter of inhibition (mm):	
			*L. monocytogenes*, 3.7–14.6; *S. aureus*, 10.9–17.6; *E. coli*, 7.5; *S.* Typhimurium, 4.5–7	
**Medicinal plants and herbs**				
*Punica granatum* L. var. *pleniflora* flowers	Ethanol	*S. aureus*, *B. cereus*, *L. monocytogenes*, *E. coli*, *S. dysenteriae*, *S. typhi*	Extract concentration: 50 mg/well	[49]
			Diameter of inhibition (mm):	
			*S. aureus*, 32; *B. cereus*, 28; *L. monocytogenes*, 32; *E. coli*, 22; *S. dysenteriae*, 30; *S. typhi*, 27	
Ziziphus and eucalyptus leaves	Aqueous and ethanol	*B. subtilis*, *E. coli*, *S. aureus*, *P. aeruginosa*, *Streptococcus sp.*	Extract concentration: 50–100 mg/mL	[50]
			Diameter of inhibition (mm):	
			*B. subtilis*, 11–19; *E. coli*, 10–16; *S. aureus*, 8–17; *P. aeruginosa*, 9–16; *Streptococcus sp.*, 11–18	
*Marsilea minuta* leaf	Methanol, hexane: methanol	*B. subtilis*, *Enterococcus faecalis*, *K. pneumoniae*, *P. aeruginosa*	MICs: from 125 to 250 µg/mL	[51]
Roselle, rosemary, clove and thyme	Aqueous and ethanol	*B. cereus*, *S. aureus*, *E. coli*, *S.* Enteritidis, *Vibrio parahaemolyticus*, *P. aeruginosa*, *Candida albicans*	MICs: from 0.313 to 20% (*w/v*)	[52]
*Pelargonium sidoides* DC.	Methanol (85%), acetone (80%)	*C. perfringens*, *S. aureus*, *Shigella flexneri*, *E. coli O157*, *S.* Typhimurium, *C. albicans*	Diameter of inhibition (mm):	[53]
			*C. perfringens*, 8–35; *S. aureus*, 13–29.7; *S. flexneri*, 13-35.3; *E. coli*, 16–36; *S.* Typhimurium, 11.3–30; *C. albicans*, 12–30	
15 Mediterranean medicinal plants	Ethanol:water (80:20)	*Camplyobacter coli*, *E. coli*, *Salmonella* Infantis, *B. cereus*, *L. monocytogenes*, *S. aureus*	Lowest MIC values (mg/mL):	[54]
			*C. coli*, 0.83 (e.g., bearberry); *E. coli*, 1.67 (bearberry); *S.* Infantis, 1.67 (bearberry); *B. cereus*, 1.67 (e.g., bearberry); *L. monocytogenes*, 1.67 (e.g., bearberry); *S. aureus*, 0.35 (bearberry)	
Ginger rhizomes	Aqueous, ethanol, *n*-hexane	*K. pneumoniae*, *S. typhi*, *Shigella* spp., *P. aeruginosa*, *E. coli*, *S. aureus*	Extract concentration: 10 µg/mL	[55]
			Diameter of inhibition (mm):	
			*K. pneumoniae*, 0.8–15.4; *S. typhi*, 13.2–16.2; *Shigella spp.*, 12.3–17.7; *P. aeruginosa*, 12.6–16; *E. coli*, 14.7–17.2; *S. aureus*, 13.3–18.3	
*Ruta chalepensis*	Methanol	*S. aureus*, *E. coli*, *P. aeruginosa*	Extract concentration: 10 mg/disk	[56]
			Diameter of inhibition (mm):	
			*S. aureus*, 12.3–16.3; *E. coli*, 13–17.3; *P. aeruginosa*, 7.7–17.7	
*Syzygium polyanthum* L. leaves	Ethanol	*E. coli* O157:H7, *K. pneumoniae*, *L. monocytogenes*, *Proteus mirabilis*, *P. aeruginosa*, S. Typhimurium, *S. aureus*, *Vibrio cholerae*, *V. parahaemolyticus*	Extract concentration: 100 µg/disk	[57]
			Diameter of inhibition (mm):	
			*E. coli*, 7; *K. pneumoniae*, 9.3; *L. monocytogenes*, 9.6; *P. mirabilis*, 6.6; *P. aeruginosa*, 7; *S.* Typhimurium, 6.6; *S. aureus*, 9.3; *V. cholerae*, 8.3; *V. parahaemolyticus*, 6.6;	
			MICs: from 0.63 to 1.25 mg/mL	
			MBCs: from 0.63 to 2.5 mg/mL	

^1^ MIC, minimum inhibitory concentration. ^2^ MBC, minimum bactericidal concentration.

**Table 3:** Antimicrobial activity of individual phenolic compounds, some examples.

Compounds	Type of Solvent	Target Organism	Antimicrobial Activity	Reference
Coumarin, quercetin	Dissolved in dimethyl sulfoxide (DMSO)	*Escherichia coli*, *Enterobacter aerogenes*, *Salmonella infantis*, *Salmonella* Typhimurium	Coumarin: MIC ^1^, 0.625–5 mg/mL; MBC ^2^, ≥ 5 mg/mL	[67]
			Quercetin: no effect	
Gallic acid, catechin	Dissolved in DMSO	*E. coli*	Inhibition haloes of 12 and 14 mm in the presence of 2.5 and 15 mg/well gallic acid and catechin, respectively.	[68]
Ellagic acid, quercetin-3-galactoside, chlorogenic acid, quercetin	Tryptic soy broth	*Listeria monocytogenes*, *Salmonella* Enteritidis	Effective concentrations: chlorogenic acid, 500 µg/mL; quercetin and quercetin-3-galactoside, 200 µg/mL; ellagic acid, 44 µg/mL	[42]
Phloridzin, phloretin	Ethanol	*Staphylococcus aureus*, *E. coli*	MIC: *S. aureus* 0.50 and 0.10 mg/mL, *E. coli* 1.50 and 0.75 mg/ml	[43]
Thymol	Dissolved in ethanol	*L. monocytogenes*	MIC: 2 mg/mL	[69]
11 phenolic compounds	Dissolved in 10% ethanol	*Bacillus subtilis*, *Bacillus cereus*, *L. monocytogenes*, *S. aureus*, methicillin-resistant *S. aureus*, *E. coli*, *S.* Typhimurium, *Pseudomonas putida*, *Pseudomonas aeruginosa*	MICs: from 125 to ≥ 500 µg/mL	[3]
			Cinnamic acid and resveratrol, 125 µg/mL; *p*-coumaric acid, 250 µg/mL; quercetin, 500 µg/mL against *B. subtilis*, *B. cereus*, respectively. Resveratrol, 250 and 500 µg/mL against *P. aeruginosa* and *P. putida*, respectively.	
17 phenolic compounds	Dissolved in absolute ethanol	19 *S. aureus* strains, including enterotoxin producers	Compound concentration: 200 µg/disk	[70]
			Hydroquinone, thymol, carvacrol, butylated hydroxyanisole, octyl gallate, and tannic acid inhibited the growth of all strains tested.	

^1^ MIC, minimum inhibitory concentration; ^2^ MBC, minimum bactericidal concentration.

**Table 4:** Antibiofilm activity of plant extracts against food pathogen microorganisms, examples from recent studies.

Source/Residue	Solvent of Extraction	Target Biofilm	Percent Biofilm Inhibition	Reference
Black Cardamom (*Amomum tsao-ko*) extract	80% ethanol	*Staphylococcus aureus*, *Salmonella* Typhimurium, *Pseudomonas aeruginosa*	45.2–51.9% (4 mg/mL cc.)	[129]
Propolis and bud poplar resins	85% ethanol	*P. aeruginosa*	50–60% (100 µg/mL cc.)	[130]
*Butia odorata* extract	acetone	*S. aureus*	99.9% (11.4–22.8 mg/mL cc.)	[131]
Onion extracts	methanol	*P. aeruginosa*, *S. aureus*	27.3–61.5% (50 µg/mL cc.)	[132]
Olive leaves	methanol	*P. aeruginosa*, methicillin-resistant *Staphylococcus aureus* (MRSA), *S. aureus*, *Bacillus subtilis*, *Escherichia coli*, *Enterococcus faecalis*, *Candida albicans*	29.3–98% (32-512 µg/mL cc.)	[133]
*Populus nigra* and *Populus alba* bud extracts	methanol	MRSA, *S. aureus*	>70% for *P. nigra*, >50% for *P. alba*	[134]
*Opuntia ficus-indica* cladodes	80% methanol	*S. aureus*	71–85% (1–1.5 mg/mL cc.)	[135]
*Eugenia* and *Syzygium* leaf extracts	acetone	*P. aeruginosa*, *S.* Typhimurium, *S. aureus*, *E. faecalis*, *E. coli*, *Bacillus cereus*	>50% for several samples (1 mg/mL cc.)	[136]
*Potentilla visianii* extracts	methanol, ethyl acetate	*Salmonella enterica*, *E. coli*, *S. aureus*, *B. subtilis*	>50% (1.1–10 mg/mL cc.)	[137]
*Gentiana asclepiadea* extracts	water, ethanol, acetone	*S. aureus*, *P. aeruginosa*, *Proteus mirabilis*	>50% (2.1–37 mg/mL cc.)	[138]

**Table 5:** Anti-staphylococcal enterotoxin effect of plant phenolic extracts and compounds.

Phenolic Extract/Compound	Target Staphylococcal Enterotoxin	Anti-Enterotoxin Activity	Reference
Licochalcone A	Enterotoxins A (SEA) and B (SEB)	Effective concentration: 0.25 mg/mL	[144]
		Secretion inhibition; Inhibition of regulatory gene (*agr*A) transcription	
Carvacrol and thymol	Not specified	Total inhibition of secretion at 0.3 and 0.15 µL/mL concentrations	[145]
Cinnamaldehyde, citronellol, eugenol, geraniol and terpineol	SEA, SEB, Enterotoxins C (SEC) and D (SED)	Concentrations: 120–1300 µg/mL	[142]
		SEA: eugenol, citronellol and geraniol reduced the production; SEB: terpineol and eugenol inhibited the production; SEC: most sensitive to the phenolics; SED: no inhibition	
16 phenolic compounds	SEA	Inhibition of SEA protein level (penta-galloyl-glucose, corilagin, punicalagin, castalagin and procyanidin B2 at 0.25 mg/mL) and activity (penta-galloyl-glucose, tannic acid, persimmon tannin, corilagin, punicalagin, eugeniin, sanguiin H-6, geraniin, pedunculagin and castalagin, 3–25 μg/mL), interaction with SEA (eugeniin, castalagin, punicalagin, pedunculagin, corilagin, geraniin, penta-galloyl-glucose and sanguiin H-6 at 0.25 mg/mL)	[146]
14 phenolic food additives	SEA	SEA production decrease: Tannic acid AL, Purephenon 50 W and Polyphenon 70A at 0.25 mg/mL; Gravinol^®^-N, Blackcurrant polyphenol AC10 and Resveratrol-P5 at 1.0 mg/mL	[147]
		Inhibition of *sea* gene expression (mg/mL): Tannic acid, 0.3; Gravinol^®^-N, 1; Blackcurrant polyphenol AC10, 1; Resveratrol-P5, 2	
Tea catechin	Enterotoxin I (SEI)	Inhibition of *sei* gene expression at 0.4 g/L concentration	[148]
Apple juice and apple polyphenols	SEA	Activity inhibition:	[149]
		Red Delicious at 0.025%	
		Apple Poly phenol-rich extract at 0.06–0.3%	
Witch-hazel and green tea extracts	SEA	Witch-hazel: inhibition of SEA production at 0.015 mg/mL GAE concentration	[143]
		Green tea: no effect	
Pomegranate extract	SEA	Inhibition of SEA production at 0.05% (*v/v*) concentration	[150]
Oleuropein	SEB	Inhibition of SEB production at > 0.2% (*w/v*) concentrations	[151]
